# Association between body roundness index (BRI) and gallstones: results of the 2017–2020 national health and nutrition examination survey (NHANES)

**DOI:** 10.1186/s12876-024-03280-1

**Published:** 2024-06-05

**Authors:** Changlong Wei, Gongyin Zhang

**Affiliations:** Department of General surgery, The First Affiliated Hospital, Jiangxi Medical College, Nanchang University, Nanchang, 330006 PR China

**Keywords:** Body roundness index, Gallstones, NHANES, Cross-sectional study, Body fat

## Abstract

**Background:**

Gallstones are associated with obesity, and the BRI is a new obesity index that more accurately reflects body fat and visceral fat levels. The relationship between BRI and gallstone risk is currently unknown, and we aimed to explore the relationship between BRI and gallstone prevalence.

**Methods:**

A cross-sectional study was conducted utilizing data from the 2017–2020 NHANES involving a total of 5297 participants. To assess the association between BRI and gallstones, we used logistic regression analysis, subgroup analysis, and interaction terms. In addition, we performed restricted cubic spline (RCS) analysis and threshold effects analysis to characterize nonlinear relationships. We assessed the ability of BRI and Body mass index (BMI) to identify gallstones using receiver operating curve (ROC) analysis and area under the curve (AUC), and compared them using the Delong test.

**Results:**

Of the 5297 participants aged 20 years and older included in the study, 575 had gallstones. In fully adjusted models, a positive association between BRI and gallstone prevalence was observed (OR = 1.16, 95% CI: 1.12–1.20, *P* < 0.0001). Individuals in the highest quartile of BRI had a 204% increased risk of gallstones compared with those in the lowest quartile (OR = 3.04, 95% CI: 2.19–4.22, *P* < 0.0001). The correlation between BRI and gallstones persisted in subgroup analyses. RCS analyses showed a nonlinear relationship between BRI and gallstones. The inflection point was further found to be 3.96, and the correlation between BRI and gallstones was found both before and after the inflection point. ROC analysis showed that BRI (AUC = 0.667) was a stronger predictor of gallstones than BMI (AUC = 0.634).

**Conclusions:**

Elevated BRI is associated with an increased risk of gallstones in the U.S. population, and BRI is a stronger predictor of gallstones than BMI. Maintaining an appropriate BRI is recommended to reduce the incidence of gallstones.

## Introduction

Gallstones are a very common digestive condition that affects about 10–20% of adults worldwide, inflicting a significant economic burden on people as well as society [1, 2]. Gallstones are formed in the gallbladder or bile ducts mainly due to unusually high cholesterol or bilirubin levels in the bile. About 80% of people with gallstones are asymptomatic, and about 20% will experience pain and complications related to gallstones [3]. In addition, if asymptomatic people do not receive timely intervention, asymptomatic people with gallstones will develop more complex diseases such as acute cholecystitis, cholangitis, and pancreatitis, which seriously affect the quality of life [4, 5]. Although prior research has identified risk factors for gallstones, solid indicators for gallstone prophylaxis are still lacking.

The causes of gallstones are multifactorial, and many risk factors have been discovered, such as obesity, metabolic syndrome, age, ethnicity, genetics, females, pregnancy, insulin resistance and diabetes, and unhealthy lifestyle habits [6–8]. Of these, obesity, particularly abdominal obesity, is highly related with the formation of gallstones [9]. Body mass index (BMI) is the most frequent metric for measuring obesity, and studies have shown that for every five-unit increase in BMI, the incidence of gallstones increases by a factor of 1.63 [10]. Nonetheless, BMI is not a reliable predictor of body fat distribution, and in 2013, Thomas et al. proposed the BRI for predicting body fat and visceral adipose tissue volume [11]. The BRI is more reflective of visceral fat and body fat percentage than previous traditional body measurements [11]. BRI has been found to be strongly linked with various diseases, however, there is still uncertainty regarding the relationship between BRI and gallstones [12–14].

The purpose of this research is to assess the association between BRI and the risk of gallstones and to provide new ideas and strategies for gallstone prevention and intervention.

## Methods

### Study design and participants

Data for this study came from NHANES between 2017 and 2020. NHANES is a stratified, multistage sample survey undertaken by the National Center for Health Statistics (NCHS) to evaluate the nutritional and health status of adults and children in the US. The NCHS ethical review board approved the survey, and each participant gave their informed consent.

**Fig. 1:**
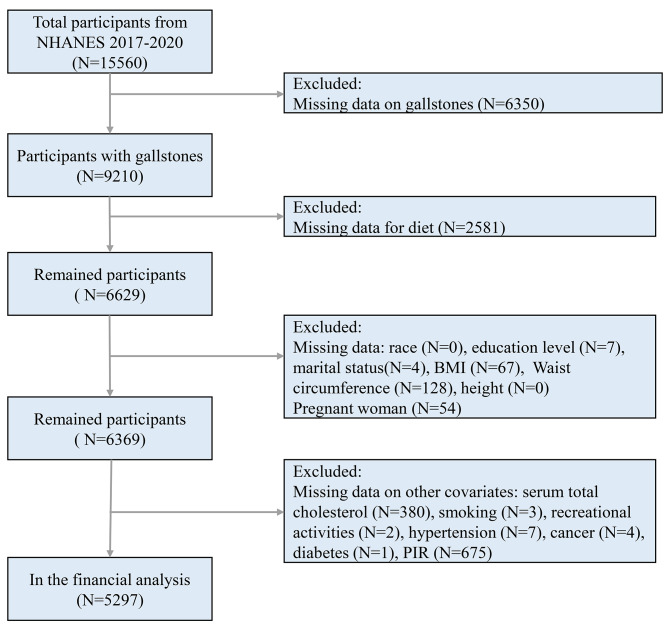
NHANES 2017–2020 Participant selection Flowchart

This study comprised 15,560 people who took part in the NHANES during January 2017 through March 2020. During this study cycle, participants were asked to provide information about their history of gallstones. To make our study more reasonable, we screened the participants, Fig. 1 illustrates the screening procedure. This study comprised 5297 people in total, 575 of whom self-reported having a history of gallstones.

### Definition of gallstones

We determined whether participants had gallstones based on the results of the questionnaire “Has doctor ever said you have gallstones”. Individuals who replied in the affirmative were classified as having gallstones, whereas those who replied in the negative were classified as not having gallstones.

### Definition of other variables

In our investigation, covariates were age, gender, race, education level, marital status, poverty-to-income ratio (PIR), recreational activities, serum total cholesterol level, smoking status, hypertension, diabetes, cancer, and dietary intake factors, which encompassed total energy intake, fat intake, sugar intake, water intake, dietary fiber intake, caffeine intake and alcohol intake. For our analysis, we used the average consumption of the two 24-hour dietary recalls for survey participants. We identified diabetes through the questionnaire “Doctor told you have diabetes”, and those who replied yes were categorized as diabetic; other similar questionnaires were used to identify hypertensive patients and cancer patients. In addition, we used the questionnaire “Smoked at least 100 cigarettes in life” to determine whether people smoked or not, and those who answered affirmatively were categorized as smokers. We obtained participants’ waist circumference (WC), height, and BMI data from the body measurements section of the NHANES database. BRI values we calculated applying the following formula [11, 15]:

$$BRI=364.2-365.5\times \sqrt{1-({\frac{wc}{2\pi })}^{2}/{\left(\frac{Height}{2}\right)}^{2}}$$

### Statistical analyses

This study’s analyses were conducted utilizing R (version 4.2.3) and EmpowerStats software (http://www.empowerstats.com). Statistical analyses were conducted utilizing appropriate NHANES sampling weights in accordance with NHANES recommendations and guidelines, accounting for the complicated multistage entire cohort survey. We investigated the association between BRI and gallstones in 3 different models using multivariate logistic regression models: model 1 was not adjusted for covariates, model 2 was adjusted for age, gender, and race, and model 3 was adjusted for all variables. We further assessed the heterogeneity between BRI and gallstones by subgroup analysis, including the following variables: age, gender, race, BMI, diabetes, hypertension, marital status, recreational activities, PIR, education level, smoking and cancer.

Furthermore, we conducted RCS analysis to evaluate the nonlinear connection between BRI and the chance of developing gallstones, with the reference value at the median. The threshold effect of BRI on the risk of gallstones was further analyzed by a two-stage linear regression model. We further evaluated the ability of BRI and BMI to identify gallstones using ROC analysis and AUC values. The AUC values of BRI and BMI were compared by Delong test [16].

## Results

### Baseline characteristics of participants

Table 1 displays the baseline characteristics of the 5,297 individuals, including 575 with gallstones and 4,722 without gallstones. We included gallstones as a column-stratified variable. In comparison to the non-gallstone group, the gallstone group had a greater proportion of females, were generally older, had higher BMI and BRI, were less likely to participate in recreational exercise, and had higher rates of smoking. Moreover, participants in the gallstone group had an increased risk of hypertension, diabetes, and cancer.

**Table 1:** Weighted baseline characteristics of participants

Characteristic	Non-gallstones (*N* = 4722)	Gallstones (*N* = 575)	*P* value
BMI (kg/m^2^), Mean (SD)	29.37 (6.66)	32.77 (8.56)	< 0.001
BRI, Mean (SD)	5.45 (2.25)	7.00 (2.97)	< 0.001
Age (years), Mean (SD)	47.33 (17.04)	57.31 (15.67)	< 0.001
PIR, Mean (SD)	3.21 (1.64)	2.95 (1.59)	0.004
Total Energy (kcal), Mean (SD)	2099.65 (832.19)	1922.11 (725.09)	< 0.001
Total sugars (gm), Mean (SD)	100.75 (62.52)	104.61 (65.78)	0.367
Total fat (gm), Mean (SD)	86.23 (39.74)	80.10 (37.40)	0.010
Water (gm), Mean (SD)	2954.97 (1287.06)	2795.72 (1239.48)	0.047
Dietary fiber (gm), Mean (SD)	16.56 (8.91)	15.50 (8.54)	0.105
Caffeine (mg), Mean (SD)	169.63 (186.27)	169.77 (164.65)	0.992
Alcohol (gm), Mean (SD)	10.76 (25.47)	5.10 (14.63)	< 0.001
Serum total Cholesterol (mg/dL), Mean (SD)	187.98 (40.56)	190.23 (41.75)	0.416
Hypertension, n (%)			< 0.001
Yes	1728 (30.6)	306 (47.9)	
No	2994 (69.4)	269 (52.1)	
Gender, n (%)			< 0.001
Male	2390 (51.3)	162 (26.3)	
Female	2332 (48.7)	413 (73.7)	
Race/ethnicity, n (%)			0.081
Mexican American	526 (8.1)	69 (7.0)	
Other Hispanic	449 (7.3)	62 (6.6)	
Non-Hispanic White	1750 (63.8)	271 (71.0)	
Non-Hispanic Black	1271 (11.0)	105 (6.7)	
Other Race	726 (9.8)	68 (8.7)	
Education level, n (%)			0.006
Less than 9th grade	250 (2.5)	21 (1.4)	
9-11th grade	480 (6.8)	64 (6.2)	
High school graduate	1096 (25.9)	144 (34.1)	
Some college or AA degree	1620 (30.7)	223 (33.3)	
College graduate or above	1276 (34.1)	123 (25.1)	
Marital status, n (%)			0.011
Married/Living with Partner	2782 (63.4)	353 (63.8)	
Widowed/Divorced/Separated	1029 (17.0)	151 (23.7)	
Never married	911 (19.6)	71 (12.5)	
Diabetes, n (%)			< 0.001
Yes	650 (10.3)	147 (19.5)	
No	4072 (89.7)	428 (80.5)	
Cancer, n (%)			0.001
Yes	472 (10.3)	101 (19.1)	
No	4250 (89.7)	474 (81.9)	
Recreational activities, n (%)			0.001
No	2350 (41.2)	342 (50.3)	
Moderate	1145 (25.9)	146 (32.0)	
Vigorous	366 (9.1)	27 (5.1)	
Both	861 (23.9)	60 (12.6)	
Smoking, n (%)			0.004
Yes	1973 (41.0)	278 (48.7)	
No	2749 (59.0)	297 (51.3)	

Categorical variables are presented as unweighted counts (weighted percentage); continuous data are presented as mean (SD).

BMI: body mass index, BRI: body roundness index, PIR: income to poverty ratio

### Associations between BRI and the risk of gallstones

Table 2 demonstrates the association between BRI and gallstone incidence. In the unadjusted model (Model 1), higher BRI was related with an increased prevalence of gallstones (OR = 1.23, 95% CI = 1.19–1.26, *P* < 0.0001). In the model adjusted for all covariates (Model 3), the positive association between BRI and gallstones persisted (OR = 1.16, 95% CI = 1.12–1.20, *P* < 0.0001). Furthermore, we converted BRI into a four-categorical variable for sensitivity assessment. In comparison to the lowest quartile of BRI, the prevalence of gallstones was increased in the second quartile (OR = 1.63,95% CI = 1.16–2.30, *P* = 0.0054), third quartile (OR = 2.27, 95% CI = 1.63–3.16, *P* < 0.0001) and fourth quartile (OR = 3.04, 95% CI = 2.19–4.22, *P* < 0.0001). The OR of gallstones increased with increasing BRI in each model (P for trend < 0.05).

**Table 2:** Association between BRI and Gallstones

	OR (95%CI), *P*-value		
	Model 1	Model 2	Model 3
BRI	1.23 (1.19, 1.26) < 0.0001	1.20 (1.16, 1.24) < 0.0001	1.16 (1.12, 1.20) < 0.0001
BRI Quartiles			
Q1, [1.258, 4.09]	1(Reference)	1(Reference)	1(Reference)
Q2, [4.09, 5.496]	1.99 (1.43, 2.78) < 0.0001	1.67 (1.18, 2.34) 0.0033	1.63 (1.16, 2.30) 0.0054
Q3, [5.496, 7.192]	3.27 (2.39, 4.47) < 0.0001	2.52 (1.82, 3.48) < 0.0001	2.27 (1.63, 3.16) < 0.0001
Q4, [7.192, 22.989]	5.14 (3.80, 6.94) < 0.0001	3.76 (2.75, 5.15) < 0.0001	3.04 (2.19, 4.22) < 0.0001
P for trend	< 0.0001	< 0.0001	< 0.0001

Model 1: no covariates were adjusted; Model 2: age, gender, and race were adjusted.; Model 3: age, gender, race, hypertension, education level, marital status, diabetes, total energy, total fat, total sugars, water, dietary fiber, caffeine, alcohol, cancer, recreational activities, smoking, serum total cholesterol, PIR were adjusted

BRI: body roundness index; PIR: income to poverty ratio; OR: odds ratio; CI: confidence interval

**Fig. 2:**
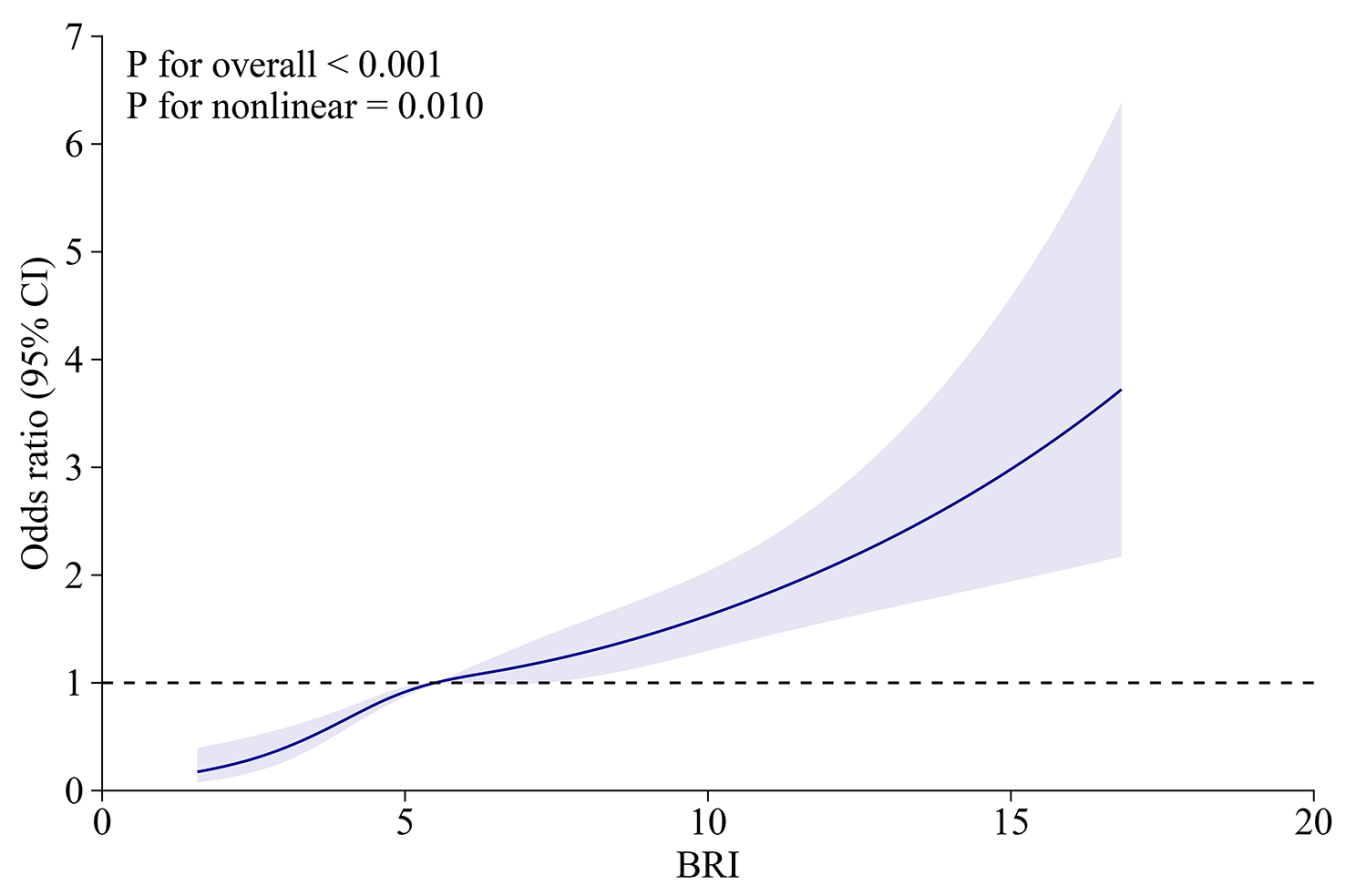
RCS analysis of the association between BRI and gallstones. Age, gender, race, hypertension, education level, marital status, diabetes, total energy, total fat, total sugars, water, dietary fiber, caffeine, alcohol, cancer, recreational activities, smoking, and serum total cholesterol were adjusted for association

The results of RCS analysis revealed (Fig. 2) that there was a significant overall trend (P for overall < 0.001) and nonlinear association (P for nonlinear = 0.010) between BRI and gallstone risk. Further threshold effect analysis showed that the inflection point for BRI was 3.96. The two-segment linear regression model showed that when BRI < 3.96 (OR = 2.25, 95% CI = 1.49–3.41, *P* = 0.0001), the risk of gallstones increased by 125% for each 1 unit increase in BRI, and 13% when BRI > 3.96 (OR = 1.13, 95% CI = 1.09–1.17, *P* < 0.0001). Table 3 shows the analysis results.

**Table 3:** Threshold effect analysis of body roundness index on gallstones using a two-piecewise linear regression model

Gallstones	Adjust OR (95% CI)	*P*-value
BRI		
Fitting by standard linear model	1.16 (1.12, 1.20)	< 0.0001
Fitting by two-piecewise linear model		
Inflection point	3.96	
<3.96	2.25 (1.49, 3.41)	0.0001
>3.96	1.13 (1.09, 1.17)	< 0.0001
Log-likelihood ratio	< 0.001	

Age, gender, race, hypertension, education level, marital status, diabetes, total energy, total fat, total sugars, water, dietary fiber, caffeine, alcohol, cancer, recreational activities, smoking, and serum total cholesterol were adjusted

### Subgroup analyses

**Fig. 3:**
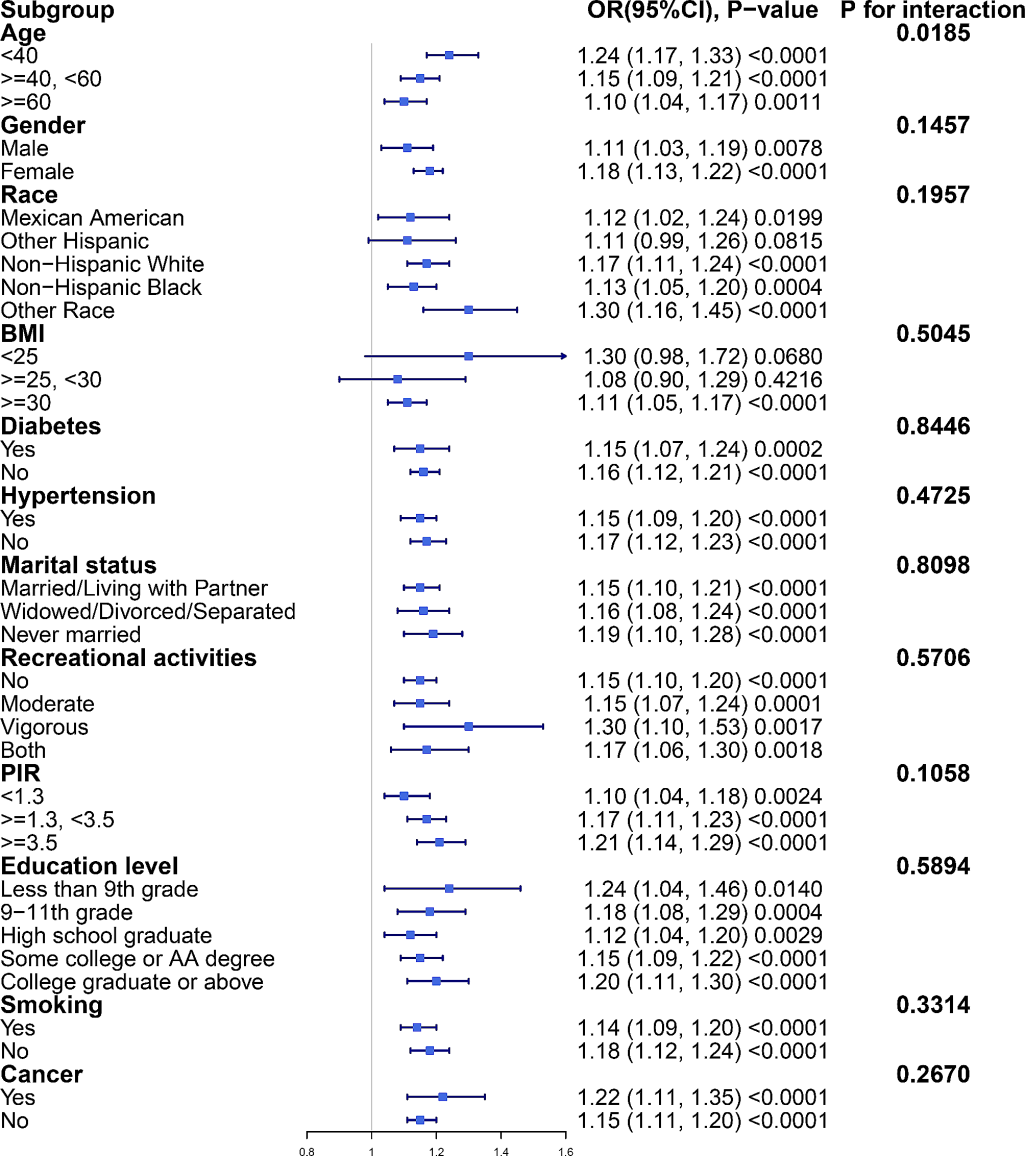
Subgroup analysis of the association between BRI and gallstones. Adjusted for all covariates except effect modifier

Figure 3 displays the subgroup analysis results. Interaction tests showed that the interaction test for the variable age was statistically significant (P for interaction = 0.0185), but in all age subgroups, BRI values were positively related with gallstone risk. There were no significant interactions in any of the other subgroups. In all subgroups, BRI values had a consistent positive correlation with gallstone risk.

### Comparison of BRI and BMI in predicting gallstones

**Fig. 4:**
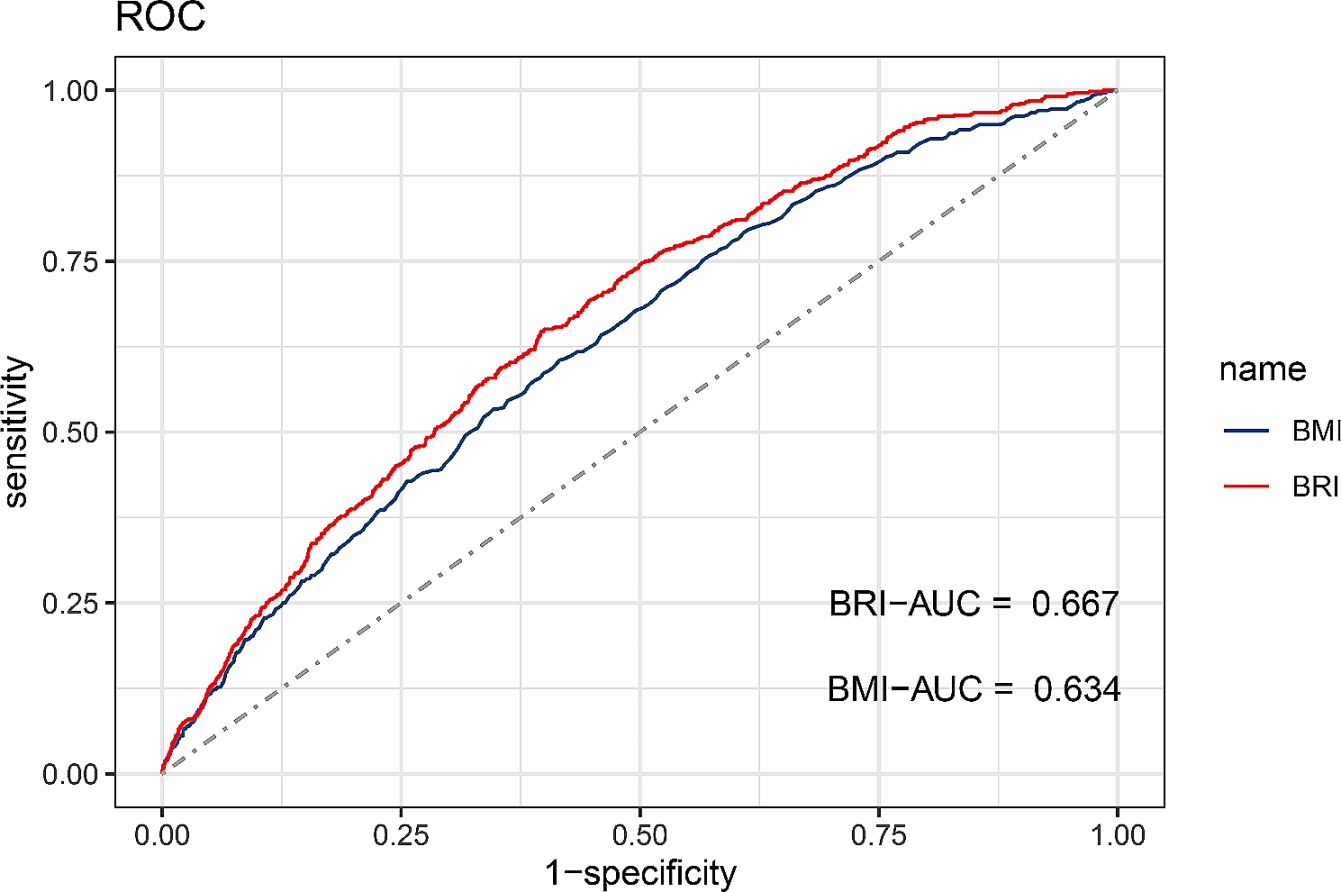
ROC curves for BRI and BMI prediction of gallstones

The results of the ROC curves are shown in Table 4; Fig. 4. ROC analysis showed that BRI (0.667) had a better AUC value than BMI (0.634) in predicting gallstones. Delong’s test showed that the difference between the AUC values of BRI and BMI was statistically significant (*P* = 1.84e-10), indicating that BRI was superior to BMI in predicting gallstones.

**Table 4:** Comparison of ROC curves for BRI and BMI in predicting gallstones

Test	AUC (95% CI)	Cutoff	Sensitivity	Specificity
BRI	0.667(0.645,0.689)	5.928	0.647	0.604
BMI	0.634(0.611,0.657)	30.2	0.605	0.584

AUC: area under the curve; CI: confidence interval

## Discussion

This cross-sectional study used data from NHANES to assess the association between BRI and gallstones. Our findings show that BRI has a positive association with an increased risk of gallstones. After adjusting for all covariates, the association remained significant. This positive correlation also persisted in the subgroup analyses. RCS showed a nonlinear relationship between BRI and gallstone risk. At BRI < 3.96, the risk of gallstones increased by 125% for each unit increase in BRI; and at BRI > 3.96, the risk of gallstones increased by 13% for each unit increase in BRI. In addition, to further explore the ability of BRI to predict gallstones, we performed a ROC analysis and compared the ability of BRI to BMI to predict gallstones. We found that BRI had a better predictive ability for gallstones than BMI and was statistically significant.

Obesity is considered an important risk factor for the formation of gallstones, and its association with gallstones has been demonstrated in many epidemiologic studies [17, 18]. Many indicators represent obesity, among which BMI, which represents overall obesity, is the most generally used index in recent years. Several research have revealed that BMI is positively linked with gallstone risk, which is consistent with our findings [19, 20]. Weight-adjusted waist circumference index (WWI) is an index for the assessment of obesity and a commonly used parameter for the assessment of obesity proposed by Park et al. This index is more reasonable and easy to assess than just BMI [21]. Similarly, higher WWI values were found to be positively linked with the prevalence of gallstones in a cross-sectional study [22]. In addition, other commonly used measures of obesity are waist-to-height ratio (WHtR), waist-to-hip ratio (WHR), waist circumference, etc. A study by Radmard et al. suggests that WHR might be the only preferable indication for estimating gallstone risk in men, and that WHtR, WHR, visceral adipose tissue thickness, and BMI are connected with the risk of gallstones in women, while subcutaneous fat was not related with the risk of gallstones [23]. However, these traditional measures of obesity have limitations. For example, BMI does not measure the specific distribution of body fat, and WC and WHtR do not distinguish between visceral and subcutaneous fat. As a result, the BRI index was created to estimate body fat percentage using waist circumference and height measurements. Several studies have shown that BRI values can accurately predict body fat and visceral adipose tissue percentages [11, 13, 24, 25]. In addition, the BRI was revealed as a predictor for the assessment of diabetes, insulin resistance, metabolic syndrome, and hyperuricemia [26–28]. Consistent with previous studies, we found that BRI can be used as a predictor for the assessment of gallstones, and that BRI values have better predictive ability compared to traditional BMI values. Our results show that BRI has higher sensitivity and specificity in predicting gallstones compared to BMI. Thus, as a newly developed obesity index, the potential of BRI as a predictor of the occurrence of gallstones is great. However, further large prospective cohort studies are still needed to confirm our opinion.

Obesity raises the risk of gallstones via multiple pathophysiologic processes. First, obesity raises insulin resistance, and leads to a variety of metabolic disorders that raise the incidence of gallstones [29]. Second, in obese individuals, cholesterol is overproduced due to upregulation of 3-hydroxy-3-methylglutaryl coenzyme A (HMG-CoA) reductase activity, which in turn promotes cholesterol gallstone formation [30]. Third, leptin is a hormone released by adipocytes. In some mouse model studies, it was found that bile cholesterol saturation decreased when mice developed hand speed resistance [31]. Obesity increases leptin secretion, leading to an excess of cholesterol secreted into the bile, which raises the risk of gallstones [32].

Our study has various strengths. First, the sample for this study came from NHANES, which has a huge sample size and reliable data. Second, we adjusted for multiple confounding variables and conducted subgroup analyses to guarantee that our findings applied to a broader group. Third, this is the first study that explores the association between BRI and gallstones, utilizing a new body measure to acquire a better understanding of the association between obesity and gallstones and finding a better potential for BRI to predict gallstones. However, our research has limitations. First, this study cannot prove a causal association between BRI and gallstones. Second, the existence or absence of gallstones in this study was determined based on a questionnaire, which implies that there may be some recall bias. Third, although we considered relevant variables, complete elimination of confounders is a major challenge. Fourth, our study failed to consider the effects of medication use and hormone levels. More relevant future studies are expected to validate our findings.

## Conclusions

Our study reveals a positive association between BRI and gallstone risk in the US adult population. In addition, BRI has better predictive ability for gallstones compared to BMI. This study aimed to increase public awareness of BRI values, a novel measure of obesity, and that maintaining a moderate BRI can help reduce the incidence of gallstones.

## Data Availability

The data used in this study are publically available in the NHANES database (www.cdc.gov/nchs/nhanes).
